# Sulforaphane Pre-Treatment Improves Alveolar Macrophage Killing After Alcohol-Induced Phagocytic Dysfunction In Vitro and in *Galleria mellonella* Larvae

**DOI:** 10.3390/medicines13010008

**Published:** 2026-02-19

**Authors:** Caleb Harrop, Nathan Clark, Robert Darby, Dallen James, Scott Quimby, Braydon Black, Vincent Tran, Ethan Ostrom, Tinna Traustadóttir, Fernando P. Monroy, Victor M. Jimenez

**Affiliations:** 1 Department of Biomedical Sciences, Noorda College of Osteopathic Medicine, 2162 S 180 E, Provo, UT 84606, USA; do25.crharrop@noordacom.org (C.H.); do26.nsclark@noordacom.org (N.C.); do27.rkdarby@noordacom.org (R.D.); do28.dmjames@noordacom.org (D.J.); do28.snquimby@noordacom.org (S.Q.); do27.brblack@noordacom.org (B.B.); do27.vqtran@noordacom.org (V.T.); 2 Department of Biological Sciences, Northern Arizona University, P.O. Box 5640, Flagstaff, AZ 86011, USA; elo42@nau.edu (E.O.); tinna.traustadottir@nau.edu (T.T.); fernando.monroy@nau.edu (F.P.M.)

**Keywords:** sulforaphane, alcohol, Burkholderia, binge drinking, MHS cells, melioidosis, *S. epidermidis*, THP-1 cells

## Abstract

**Background:** Alcohol is associated with increased mortality and morbidity globally. Pulmonary infections with opportunistic pathogens can occur in healthy humans; however, binge alcohol intoxication (≥0.08% BAC) is a major risk factor. We have previously shown that a single dose of alcohol comparable to binge alcohol intoxication increases infection by reducing alveolar macrophage function in vivo. Sulforaphane (SFN), a phytonutrient, is a potent inducer of antioxidant production through the induction of nuclear factor erythroid 2-related factor 2 (Nrf2) and inhibition of the nuclear factor kappa-light-chain-enhancer (NF-kB) pathway. The aim of this study was to test the therapeutic potential of SFN given as a pretreatment to prevent alcohol-induced phagocytic dysfunction. **Methods:** Intracellular phagocytic killing was measured via colony-forming units (CFU) and cytokine expression via ELISA. *G. mellonella* survival was used to determine the therapeutic potential of SFN in vivo. **Results:** Dose–response curves indicated that SFN concentrations of less than 20 µM were not cytotoxic in either MH-S (murine) or THP-1 (human) cells. Live infection assay results showed that MH-S and THP-1 cells pretreated with SFN (5 µM) and challenged with 0.2% (*v*/*v*) alcohol for 3 or 8 h prior to live *B. thailandensis* or *S. epidermidis* infection improved intracellular pathogen killing between 12- and 20-fold compared to macrophages treated with alcohol alone. ELISA analysis indicated that SFN significantly reduced levels of Tumor necrosis factor-alpha (TNF-α) expression at 3 and 8 h compared to controls. Additionally, a *Galleria mellonella* larvae model demonstrated greater survivability in the prophylaxis group compared to larvae exposed to either Gram-positive or Gram-negative pathogens, as well as in groups that received alcohol prior to pathogen inoculation. **Conclusions:** Taken together, SFN-induced cytoprotection was extended beyond in vitro cell culture to include an in vivo *G. mellonella* model demonstrating protection against Gram-positive and negative opportunistic pathogens. These data demonstrate that SFN may be an effective pretreatment option to prevent alcohol-mediated innate immune dysfunction and restore macrophage phagocytic killing.

## 1. Introduction

Alcohol is one of the most abused drugs worldwide [1,2]. Hazardous alcohol consumption is commonly associated with liver, brain, and gastrointestinal tract disorders; however, since the COVID-19 pandemic, diseases of the respiratory system are becoming more widely linked to hazardous alcohol consumption [3]. Ties between chronic alcohol abuse and pneumonia have been well established for some time [4]. However, the connection between binge alcohol intoxication and pulmonary bacterial infections has not been described until more recently [5,6,7]. Binge alcohol intoxication is a hazardous alcohol intake pattern characterized by the consumption of 4–6 standard drinks or reaching a minimum of 0.08% or higher alcohol concentration within a 2–3 h episode [8]. Hazardous alcohol consumption, but not necessarily a binge intake pattern, has been shown to alter the initial host–pathogen interactions during infections caused by *Mycobacterium avium* Complex (MAC)*, Escherichia coli*, *Streptococcus pneumoniae*, *Klebsiella pneumoniae*, *Staphylococcus aureus*, and *Burkholderia thailandensis* [9,10,11,12]. The exact mechanisms by which alcohol can lead to pulmonary pathology remain unclear, but a major focal point is innate immune dysfunction [13,14]. Alveolar macrophages (AMs) are the first line of defense during pulmonary infections, located in the distal respiratory tract, and are critical for detecting, capturing, and eliminating pathogens while initiating the early host immune response. It has been proposed that chronic alcohol exposure, in conjunction with disruptions in the alveolar epithelial barrier integrity and alterations in host defenses of the upper and lower airways, induces a phenotypic change in alveolar macrophages, collectively labeled the “alcoholic lung” phenotype. Chronic alcohol exposure also inhibits the expression and function of nuclear factor erythroid 2-related factor 2 (Nrf2), preventing Nrf2-dependent production of antioxidants crucial to fighting against pulmonary infections [15].

Opportunistic pathogens, such as *B. thailandensis* and *S. epidermidis,* take advantage of impaired early host immune responses to cause infection [16]. Growing antibiotic resistance makes these types of opportunistic infections a source of great concern [17]. *S. epidermidis* is a Gram-positive bacterium that is associated with pneumonia in immunocompromised individuals [18,19,20,21]. *B. thailandensis* is a Gram-negative bacterium found in the environment. Both *B. thailandensis* and *B. pseudomallei* are endemic to the regions of northern Australia and southeast Asia. However, *B. thailandensis* has been detected in the United States in the areas of Puerto Rico, Mississippi, and southern Texas by water and soil sampling [22]. A single binge-like alcohol dose altered phagocytosis in murine AM cells and increased the intracellular survival of *B. thailandensis* in vitro [23]. Additionally, in vivo, *B. thailandensis* infectivity increased in mice exposed to a binge-like alcohol dose, thus disseminating bacteria in the bloodstream and colonizing major organs [24].

Various hospital treatments for hazardous alcohol consumption exist; however, a viable prophylactic option to mitigate the toxic effects of hazardous alcohol consumption and protect otherwise healthy individuals from opportunistic infections is currently unavailable and not well studied. Phytonutrients are a class of plant-derived compounds that are bioactive and can affect cytoprotective cellular processes. One of the more promising phytonutrients is sulforaphane (SFN), an isothiocyanate, generated from the enzymatic cleavage of glucoraphanin, which is present in cruciferous vegetables such as kale and broccoli [25]. Although SFN itself is weakly prooxidant, SFN has been shown to induce cytoprotective measures via Nrf2. Nrf2 is a redox-sensitive transcription factor that regulates the activation of the antioxidant response element (ARE), which has a significant role in cellular protection via antioxidant and detoxifying enzymes [26,27,28]. Furthermore, SFN protects against the effects of oxidative damage and phagocytic dysfunction induced by chronic alcohol use in alveolar macrophages [29]. Phagocytic activity and engulfment have been shown to increase in mouse RAW 264.7 macrophages when pretreated with SFN in the absence of an alcohol stimulus [30]. Given this evidence, SFN is attractive for therapeutic purposes to prevent alcohol-induced innate immune dysfunction in the context of infection because of its cellular protective properties, and the compound requires a low concentration for maximal bioactivity [26].

Small-molecule drug discovery is a multi-step process that integrates target validation, fragment-based screening, followed by lead optimization and preclinical testing. In addition to basic toxicology cell culture studies, low-cost insect models can significantly inform compound selection and early toxicity profiling, leading to fewer failures in rodent models and ultimately, human trials. *Galleria mellonella* larvae, also known as wax worms, were first used for published research in the 1950s [31]. They are a useful bridge between cellular and mammalian experiments because they have a notably similar innate immune system to that of mammalian species [32]. As such, they have been widely used in studying bacterial and fungal infections and have served as low-cost models for testing new antibiotic therapies [33]. An additional benefit to the *G. mellonella* model for the study of human opportunistic infection is the standard 37 °C incubation. This temperature induces expression of bacterial virulence factors and is at or near human body temperature [31,34], thus creating a more relevant clinical context for research.

The present study evaluated SFN’s therapeutic potential against alcohol-induced immune dysfunction using both in vitro and in vivo models. First, we established dose–response curves in murine (MH-S) and human (THP-1) macrophage cell lines. Next, we assessed SFN as a prophylactic agent by pretreating these cells prior to exposure to hazardous levels of alcohol. We then extended our in vitro findings by challenging SFN-pretreated macrophages with live bacterial infection. Finally, we translated this paradigm into an in vivo *G. mellonella* larval model, measuring survival after SFN pretreatment and subsequent alcohol and bacterial challenge. We hypothesized that SFN pretreatment would preserve macrophage phagocytic function previously shown to be impaired by alcohol and improve larval survival compared to controls.

## 2. Materials and Methods

### 2.1. Cell Culture

Murine alveolar macrophage (ATCC: MH-S, CRL-2019) and human monocytes (ATCC: THP-1, TIB-202) were used in these studies at passages ≤6 and ≥90% confluency. Cells were regularly grown in T-75 cell culture flasks in phenol red-free RMPI-1640 medium (Gibco, Life Technologies, Bleiswijk, The Netherlands) supplemented with 10% fetal bovine serum, 2 mM L-glutamine, 10 mM HEPES, 0.1 mM non-essential amino acids, 1.5 g/L sodium bicarbonate, 50 U/mL penicillin, and 50 mg/mL streptomycin. Both MH-S and THP-1 cells were incubated at 37 °C and 5.5% CO_2_. Cells were seeded in 24-well cell culture plates at 1 × 10^6^ cells/well and incubated for all experiments in low-evaporative cell culture plates, a compensating system previously described by Jimenez et al. to avoid the evaporation of alcohol during treatments [23]. RPMI-1640 media supplemented with alcohol (ethanol absolute, 200 proofs, Fisher BioReagents, Waltham, MA, USA) were used based on biological relevance and cell viability > 85%.

THP-1 cell differentiation: THP-1 cells required differentiation from monocytes to macrophages prior to SFN and alcohol exposure. This was accomplished by incubating THP-1 cells in 100 ng/mL of PMA (phorbol myristate acetate, Cayman Chemical Item No. 10008014, Ann Arbor, MI, USA) and 10 ng/mL Interferon-gamma (IFN-γ) for 24 h and then verified using microscopy. After differentiation, cells were rinsed and immediately used experimentally.

### 2.2. Cell Viability Assay

To optimize the dose of SFN or alcohol, dose–response experiments were conducted. Both MH-S and THP-1 cells were cultured with 0, 2.5, 5, 10, 20, or 50 μM SFN for 2 or 24 h. The effects of alcohol (i.e., ethanol) on MH-S and THP-1 cell viability were tested in a similar procedure; cells were cultured with 0, 0.08, 0.2, 0.4% alcohol (*v*/*v*) for 3 or 24 h. Five µM SFN and 0.2% (*v*/*v*) alcohol concentrations were used for all further experiments based on ≥85% cell viability after a CyQUANT LDH viability assay (ThermoFisher Cat # C20300, Waltham, MA, USA). Briefly, spontaneous, positive, vehicle controls, and samples were transferred to a flat-bottom 96-well plate, incubated with the reaction mixture, and read via BioTek Synergy microplate reader (BioTek Instruments, Winooski, VT, USA) at wavelengths described by the manufacturer.

The percentage of viable cells was calculated as follows:

Viable Cells (%) = 100% − ((Compound-treated LDH − Spontaneous LDH)/(Max LDH − Spontaneous LDH) × 100))

### 2.3. Intracellular Killing Assay

Both MH-S and differentiated THP-1 cell lines were pre-treated with 0 or 5 μM SFN for 24 h; cell monolayers were rinsed and then challenged with 0 or 0.2% (*v*/*v*) alcohol. Cell monolayers were co-cultured with viable *B. thailandensis* for MH-S cells, or *S. epidermidis* for THP-1 cells at a multiplicity of infection (MOI) of 1:1 for 3 h and 8 h at 37 °C, and with 5.5% CO_2_, to allow phagocytosis to occur. At an MOI of 1:1, cell viability was ≥90%. After 3 and 8 h, extracellular bacteria were removed by washing the cells with DPBS and replacing the culture media with new media supplemented with 250 μg/mL of kanamycin for 1 h. Thereafter, the cell monolayers were incubated at 37 °C in media with 50 μg/mL kanamycin for an additional 2 h to completely remove any residual extracellular bacteria. Any intracellular bacteria remained unaffected by the antibiotic treatment.

Intracellular phagocytic killing occurred for a total of 3 h post-infection. After 3 h, the cell culture media was removed, and the infected cell monolayers were washed. To determine the ability of MH-S and THP-1 cells to kill intracellular bacteria, cell monolayers were lysed with DPBS containing 0.1% Triton X-100. Viable intracellular bacteria were quantified by plating serial dilutions of the lysate, and average CFUs were determined on LB agar plates.

### 2.4. Enzyme-Linked Immunosorbent Assay (ELISA)

Samples for the ELISA were prepared using extracted supernatant from the intracellular killing assay for human THP-1 cells from both the 3 h and 8 h cultures prior to the initial DPBS wash. Invitrogen ELISA kits were used to measure levels of TNF-α (Cat #88-7346-22), Interleukin-10 (IL-10) (Cat #88-7106-22), and IFN-γ (Cat #88-7316-22) in accordance with the manufacturer’s instructions. Testing was run in triplicate in a 96-well plate for each condition collected from the human THP-1 experiment. The controls were plated in duplicate. Absorbance measurements were quantified by the BioTek synergy microplate reader at wavelengths described by the manufacturer.

### 2.5. Galleria mellonella Model

*Galleria mellonella* larvae were purchased from Bio suppliers (Daejeon, Korea), maintained at 37 °C in the dark, and used within 3 days. The normal supply of larvae was fasted prior to experimental use. Larvae weighed between 100 and 150 mg at the time of inoculation. Twenty larvae per group (N = 20) were each injected with 10 μL of inoculum into the caudal prolegs using a 22s-gauge point style 25 µL microliter syringe (Hamilton, Reno, NV, USA, PN 84855). Each group of 20 insects was incubated at 37 °C in 9 cm Petri dishes up to 5 days unless otherwise specified.

### 2.6. Galleria mellonella Survival Curves

**Figure 1:**
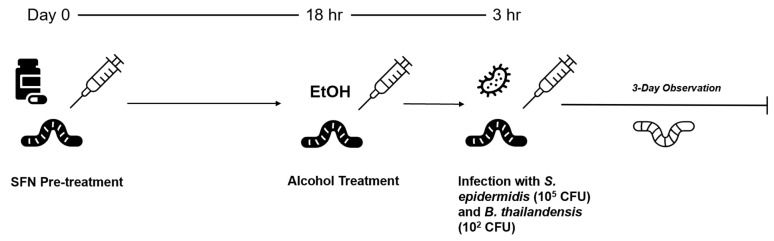
*G. mellonella* larvae were injected on day 0 with an SFN pre-treatment in the caudal prolegs. Eighteen hours later, larvae received injections of alcohol, followed three hours later by inoculation with *B. thailandensis* or *S. epidermidis*. The larvae were monitored over the course of three days for changes in activity and color.

Survival curves were generated to optimize doses of SFN, alcohol, *S. epidermidis*, and *B. thailandensis* in the larvae. For the prophylaxis study, the *G. mellonella* larvae were divided into nine test groups. The groups consisted of the following: negative control (no injections), vehicle control (1% DMSO), and the rest of the groups were inoculated with 100 µM SFN, 0.08% (*v*/*v*) ALC, and/or *S. epidermidis* or *B. thailandensis*. SFN was injected into the right caudal proleg, ALC in the left caudal proleg, and bacteria in the right cephalad proleg to minimize injection trauma. The syringes were cleared with either PBS, 1% DMSO, or LB broth after each larva was injected, depending on the solvent used for SFN, ALC, and pathogens. Injections were spaced so that ALC was administered 3 h after SFN prophylaxis, and then with pathogen inoculation 1 h later (Figure 1).

### 2.7. Monitoring and Infection of Galleria mellonella Larvae

To optimize doses for SFN, alcohol, or to study various post-infection doses, *G. mellonella* larvae were monitored over a 72 h period in 24 h intervals for the following attributes: activity, extent of silk production (cocoon formation), and melanization. The attributes were used to determine survival. In addition, a score was provided that contributed toward an overall health index of an individual wax worm. A healthy wax worm typically scored between 9 and 10, and an infected, dead wax worm typically scored 0. For infections, a sterile 20 μL Hamilton syringe (30 gauge) was used to inject 10 μL aliquots of bacterial suspensions in PBS into the prolegs of *G. mellonella*. Larvae were infected with various CFU/larvae, with a minimum of 20 larvae per group. The control group was injected with 10 μL phosphate-buffered saline (PBS) (Life Technologies). Following the injections, larvae were incubated at 37 °C in the dark to allow infection. Every 24 h, larvae were scored as dead or alive as previously described. To control the precise bacterial load per injection, larvae were infected with bacterial strain, and at fixed time points after infection (1, 24, 48, and 72 h p.i.), alive larvae were bled into microcentrifuge tubes, and bacterial burden was determined using the viable plate count method with LB agar plates.

### 2.8. Statistical Analysis

The data analysis was completed using Prism 10.0 software (Graph Pad, 10.1, San Diego, CA, USA). The assay replicating independence and significance was determined by two-way ANOVA with Bonferroni multiple comparisons and Student’s *t*-test. Each N represents at least 4 experimental replicates (i.e., identical experimental wells) and at least 2 assay replicates from each experimental replicate. Each cell culture experiment was conducted independently at least twice on different days. Survival analysis of *G. mellonella* consisted of Kaplan–Meier survival curves (log-rank test). At least three independent experiments were conducted, and representative curves are represented on the plot. A *p*-value ≤ 0.05 was considered significant (N = 20).

## 3. Results

### 3.1. Cytotoxic and Temporal Effects of SFN and Alcohol Are Similar in MH-S and THP-1 Cells

**Figure 2:**
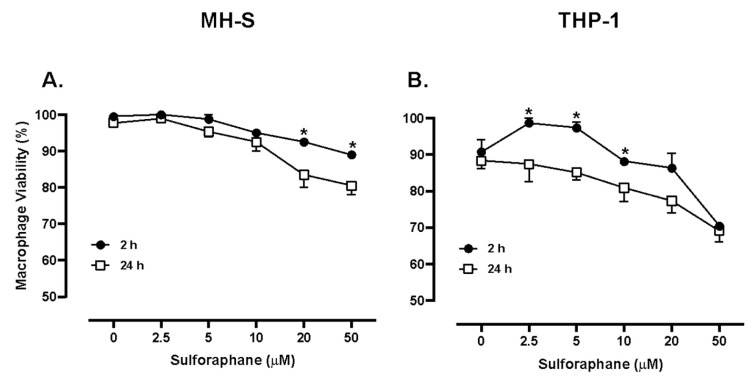
MH-S cell viability (**A**) and THP-1 cell viability (**B**) when exposed to varying concentrations of SFN. Murine MH-S and human THP-1 cell monolayers were treated with media supplemented with 0, 2.5, 5, 10, 20, or 50 µM SFN. MH-S and THP-1 cells were treated for 2 or 24 h. N = 4. Data are expressed as means ± SEM. Statistically significant results by one-way ANOVA are indicated by asterisks * *p* < 0.05.

Cell viability was not significantly decreased at 2 and 24 h in MH-S cells supplemented with 2.5, 5, or 10 μM SFN. A decrease of approximately 5 or 10% in cells treated with 20 or 50 μM SFN, respectively, was recorded after 2 h; also, a significant decrease of approximately 20% viability was recorded in MH-S cells treated with 20 or 50 μM SFN for 24 h (Figure 2A). Similarly, THP-1 cell monolayers were treated with 0, 2.5, 5, 10, 20, or 50 µM SFN. Cell viability was increased at doses 2.5 and 5 µM SFN; however, a trend toward a dose-dependent decrease in viability remained at 10, 20, and 50 µM SFN at 2 h incubation. THP-1 cell viability significantly decreased at doses 2.5, 5, and 10 µM SFN when compared to the 24 h incubation. Cell viability decreased approximately 20% when treated with 50 µM SFN at both 2 and 24 h (Figure 2B).

**Figure 3:**
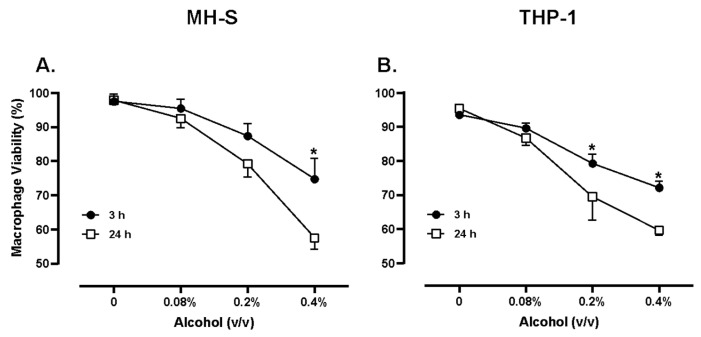
MH-S cell viability (**A**) and THP-1 cell viability (**B**) when exposed to varying concentrations of alcohol. Murine MH-S and human THP-1 cell monolayers were treated with media supplemented with 0.08%, 0.2%, and 0.4% alcohol (*v*/*v*) for 3 or 24 h. N = 4. Data are expressed as means ± SEM. Statistically significant results by one-way ANOVA are indicated by asterisks * *p* < 0.05.

Also, dose–response experiments were conducted to test the cytotoxicity of increasing alcohol (ALC) dosage on MH-S and THP-1 cells; alcohol was tested in an analogous manner using concentrations of 0.08%, 0.2%, and 0.4% for 3 and 24 h exposures. Viability significantly decreased in MH-S cells exposed to media supplemented with 0.4% (*v*/*v*) alcohol for 3 or 24 h. A dose-dependent decrease in MH-S cell viability was recorded (Figure 3A). Viability significantly decreased in THP-1 cells exposed to media supplemented with >0.2% (*v*/*v*) alcohol for 3 or 24 h. A dose-dependent decrease in THP-1 cell viability was recorded (Figure 3B).

### 3.2. SFN Pre-Treatment Prevents Phagocytic Intracellular Killing Dysfunction

**Figure 4:**
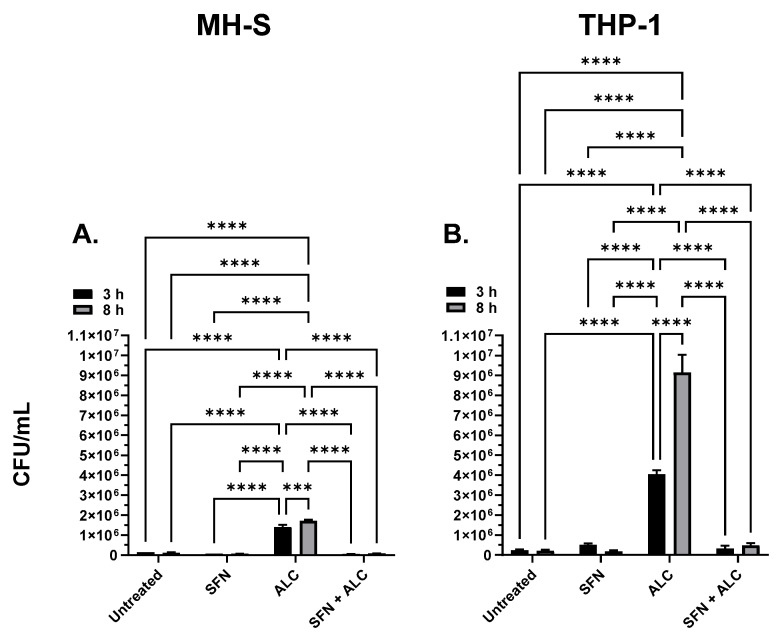
MH-S cell intracellular killing (**A**) and THP-1 intracellular killing (**B**). Murine MH-S and human THP-1 cell monolayers were pretreated with media supplemented with 5 μM SFN for 24 h, rinsed and challenged with 0.2% alcohol (*v*/*v*) for 3- or 8 h; bacterial infection occurred concurrently for 3 or 8 h. Groups represent the following: untreated = baseline phagocytosis, no sulforaphane (SFN), no alcohol (ALC); SFN = cells pretreated with SFN, no alcohol; ALC = cells challenged with ALC, no SFN; SFN + ALC = cells pretreated with SFN and challenged with ALC. All groups were challenged with live bacteria. N = 8. Data are expressed as means ± SEM. Statistically significant results by two-way ANOVA indicated by asterisks *** *p* < 0.001, **** *p* < 0.0001.

To test the prophylactic potential of SFN in the context of hazardous alcohol (ALC) exposure, MH-S and THP-1 cells were pretreated with 5 μM SFN for 24 h and challenged with a binge-like alcohol dose 0.2% (*v*/*v*) for 3 or 8 h. *B. thailandensis* intracellular survival was significantly increased in MH-S cells treated with 0.2% (*v*/*v*) alcohol for 3 and 8 h in the absence of SFN. MH-S cells pretreated with SFN and then challenged with alcohol significantly decreased *B. thailandensis* intracellular survival approximately 20-fold compared to cells treated with alcohol alone (Figure 4A). No significant difference was recorded for cells pretreated with SFN plus the alcohol challenge compared to the SFN control or untreated control (i.e., no SFN and no alcohol). Similarly, *S. epidermidis* intracellular survival was significantly increased in THP-1 cells treated with 0.2% (*v*/*v*) alcohol for 3 and 8 h in the absence of SFN. THP-1 cells pretreated with SFN and then challenged with alcohol significantly decreased *S. epidermidis* intracellular survival approximately 18-fold compared to cells treated with alcohol alone (Figure 4B). No significant difference was recorded for cells pretreated with SFN plus the alcohol challenge, compared to SFN control or untreated control (i.e., no SFN and no alcohol).

### 3.3. SFN Treatment Prior to Alcohol Exposure and Bacterial Challenge Decreases TNF-α

**Figure 5:**
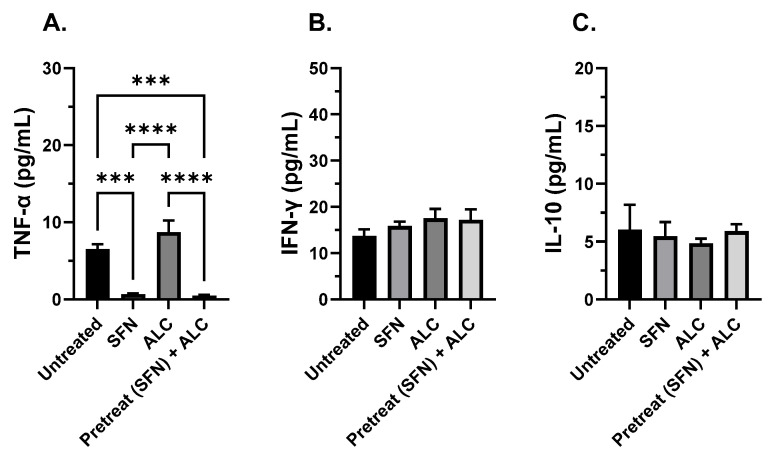
Cytokine expression after 3 h alcohol exposure. TNF-α (**A**), IFN-γ (**B**), and IL-10 (**C**). Human THP-1 cell monolayers were pretreated with media supplemented with 5 μM SFN for 24 h, rinsed, and challenged with 0.2% alcohol (*v*/*v*) and co-cultured with live *S. epidermidis*. N = 4. Groups represent the following: untreated = baseline expression, no sulforaphane (SFN), no alcohol (ALC); SFN = cells pretreated with SFN, no alcohol; ALC = cells challenged with ALC, no SFN; SFN + ALC = cells pretreated with SFN and challenged with ALC. Data are expressed as means ± SEM. Statistically significant results by one-way ANOVA are indicated by asterisks *** *p* < 0.001, **** *p* < 0.0001.

To measure the immunomodulating effects of SFN in the human condition, the supernatant from human THP-1 cells was collected from all four groups described previously (Figure 4) at 3 and 8 h. Samples were processed through an ELISA to test for changes in levels of pro-inflammatory TNF-α, IFN-γ, and anti-inflammatory IL-10 cytokines. THP-1 cells pre-treated with SFN, then challenged with alcohol for 3 h and co-cultured with live *S. epidermidis,* significantly decreased TNF-α levels compared to cells exposed to alcohol alone or untreated controls. Cells treated with SFN only significantly decreased TNF-α compared to alcohol and untreated controls. No significant difference was recorded between alcohol alone and untreated groups at 3 h incubation. (Figure 5A). No significant differences were recorded between groups when IFN-γ or IL-10 cytokines were tested (Figure 5B,C).

**Figure 6:**
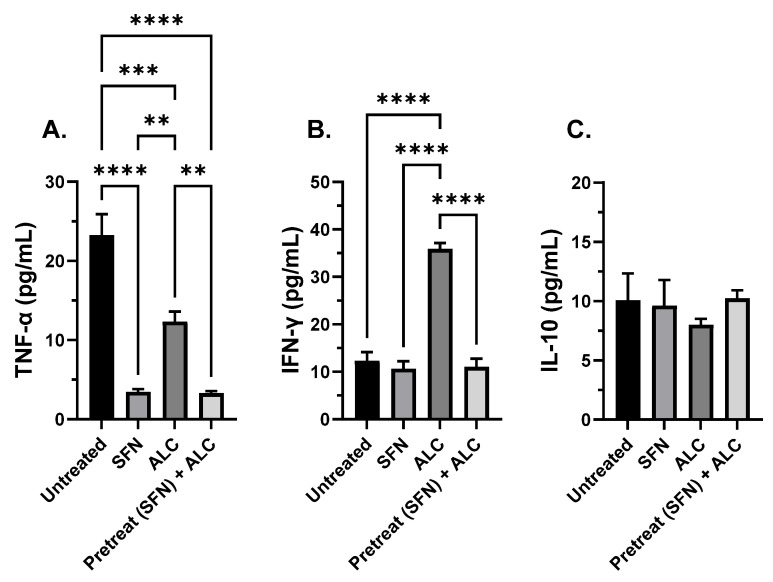
Cytokine expression after 8 h alcohol exposure. TNF-α (**A**), IFN-γ (**B**), and IL-10 (**C**). Human THP-1 cell monolayers were pretreated with media supplemented with 5 μM SFN for 24 h, rinsed and challenged with 0.2% alcohol (*v*/*v*) and co-cultured with live *S. epidermidis*. N = 4. Groups represent the following: untreated = baseline expression, no sulforaphane (SFN), no alcohol (ALC); SFN = cells pretreated with SFN, no alcohol; ALC = cells challenged with ALC, no SFN; SFN + ALC = cells pretreated with SFN and challenged with ALC. Data are expressed as means ± SEM. Statistically significant results by one-way ANOVA are indicated by asterisks ** *p* < 0.01, *** *p* < 0.001, **** *p* < 0.0001.

THP-1 cells pretreated with SFN, then challenged with alcohol for 8 h and co-cultured with live *S. epidermidis,* significantly decreased TNF-α levels compared to cells exposed to alcohol alone or untreated controls. Cells treated with SFN only significantly decreased TNF-α compared to alcohol and untreated controls. Untreated cells significantly increased TNF-α levels compared to all other groups. Alcohol alone significantly decreased TNF-α compared to untreated controls after 8 h incubation (Figure 6A). Similarly, cells pretreated with SFN, then challenged with alcohol for 8 h and co-cultured, significantly decreased IFN-γ levels compared to cells exposed to alcohol alone. Untreated and SFN only groups significantly decreased compared to the alcohol alone group; no significant difference was recorded between pretreatment plus alcohol challenge, and SFN alone, untreated groups (Figure 5B). No significant differences were recorded between groups when IL-10 was tested (Figure 6C).

### 3.4. Selected SFN Doses Demonstrate High Tolerance in G. mellonella Larvae Model

**Figure 7:**
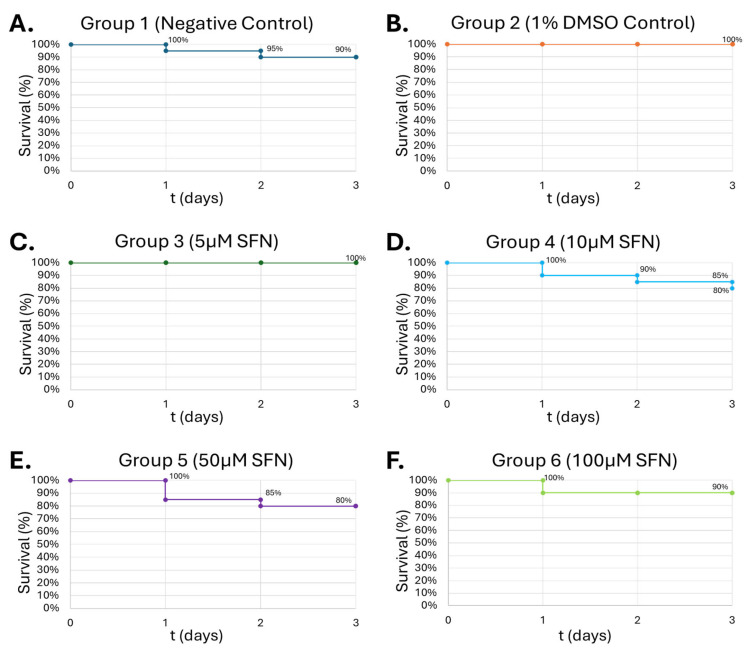
**Survival of *G. mellonella* larvae after treatment with different concentrations of SFN.** Negative control (**A**) = no injection, vehicle control (**B**) = vehicle, 5 µM (**C**), 10 µM (**D**), 50 µM (**E**), 100 µM (**F**). Day zero represents the actual day of the inoculation, with subsequent days representing 24 h periods. N = 20. No statistical difference was found between groups.

*G. mellonella* larvae are especially useful in studying the relationship between drug compounds, infections, and the innate immune system. The negative control group exhibited a 10% mortality (Figure 7A) while the vehicle control (1% DMSO) maintained 100% survival over the 72 h observation period (Figure 7B), confirming that neither the injection procedure nor DMSO had toxic effects. Low-dose SFN (5 µM) was well tolerated, with 100% survival throughout the experiment (Figure 7C). Groups exposed to 10 µM and 50 µM SFN both showed reduced survival over time (Figure 7D,E). Survivability with 10 µM SFN declined to 90% in 24 h, 85% by 48 h, and down to 80% by the end of the 72 h observation period. Survivability to 50 µM SFN dropped to 85% in 24 h, then declined to 80% by 48 h.

### 3.5. Alcohol Insult in G. mellonella Larvae Demonstrates Dose-Dependent Survival with Increasing Dose

**Figure 8:**
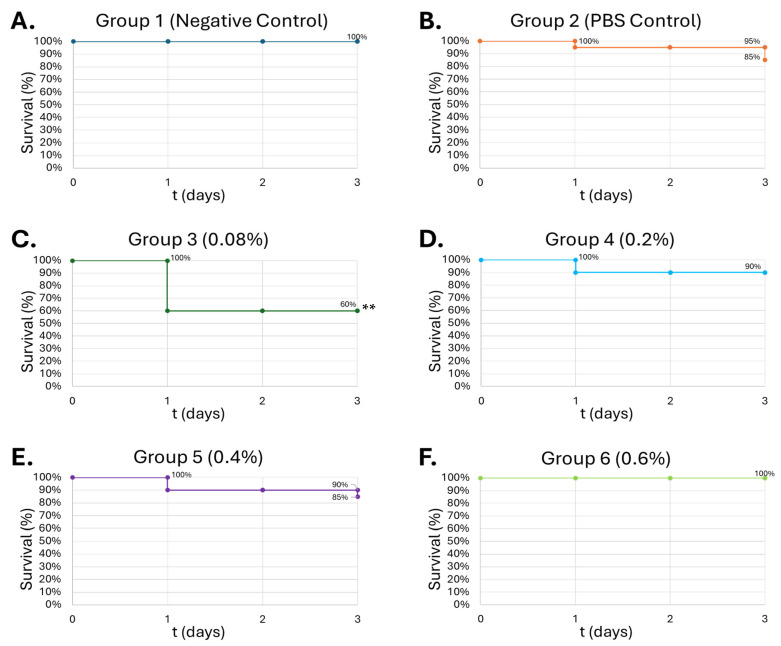
**Survival curve of *G. mellonella* larvae (n = 20) after 10 µL of increasing ALC concentration.** Negative control (**A**) = no injection, PBS control (**B**) = vehicle, 0.08% (*v*/*v*) (**C**), 0.2% (*v*/*v*) (**D**), 0.4% (*v*/*v*) (**E**), 0.6% (*v*/*v*) (**F**). Day zero = injection day. N = 20. Asterisks represent statistical differences between untreated control groups and treated groups (survival analysis, *p* < 0.01, **).

Alcohol (10 µL) was introduced into *G. mellonella* larvae at increasing concentrations. Concentrations were chosen to simulate differing BAC in humans during binge-like alcohol exposure. Larvae injected with PBS solution demonstrated 90% survivability after 72 h of observation. The 0.08% (*v*/*v*) alcohol group showed the greatest lethality among all groups, with a 60% survival rate (Figure 8C). Increasing ALC concentration demonstrated greater survivability, up to 100% survival at 0.6% (*v*/*v*) (Figure 8D–F).

### 3.6. Pathogen Insult in G. mellonella Larvae Demonstrates Dose-Dependent Lethality

**Figure 9:**
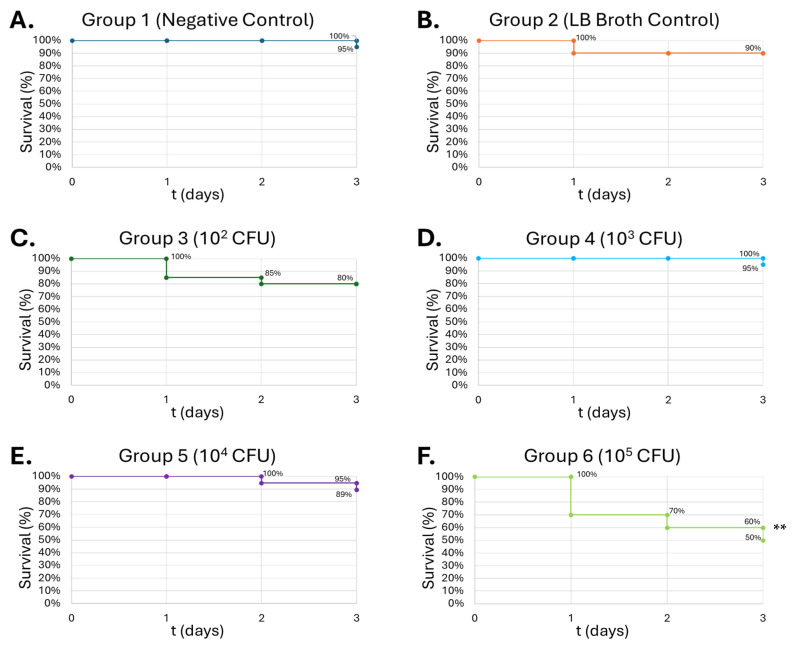
**Survival of *G. mellonella* larvae (n = 20) after different doses of *S. epidermidis*.** Negative control (**A**) = no injection, LB Broth control (**B**) = vehicle control, 10^2^ CFU (**C**), 10^3^ CFU (**D**), 10^4^ CFU (**E**), 10^5^ CFU (**F**). Day zero = injection day. N = 20. Asterisks represent statistical differences between untreated control groups and treated groups (survival analysis, *p* < 0.01, **).

The negative control and LB broth groups had minimal mortality in the *G. mellonella* larvae. The negative control exhibited 95% survival, and the LB broth vehicle control 90% survival (Figure 9A,B). Group 3 (10^2^ CFU) exhibited 80% survivability over a 72 h period, during which three deaths occurred on the first day, followed by one death on the second day (Figure 9C). Inoculation with 10^3^ CFU exhibited one death on the third day of the 72 h period (Figure 9D). Similarly, 10^4^ CFU exhibited two deaths over the 72 h period: one during the second day and the other during the third day (Figure 9E). Inoculation with 10^5^ CFUs exhibited the highest mortality rate within the *S. epidermidis* groups, with six deaths on the first day, two deaths on the second day, and two more deaths on the third day, resulting in 50% survival.

**Figure 10:**
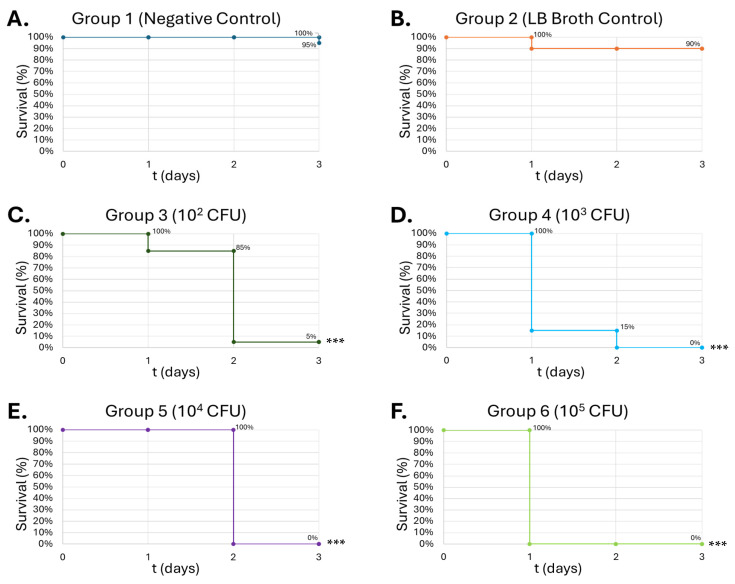
**Survival of *G. mellonella* larvae (n = 20) after different doses of *B. thailandensis*.** Negative control (**A**) = no injection, LB Broth control (**B**) = vehicle control, 10^2^ CFU (**C**), 10^3^ CFU (**D**), 10^4^ CFU (**E**), 10^5^ CFU (**F**). Day zero = injection day. N = 20. Asterisks represent statistical differences between untreated control groups and treated groups (survival analysis, *p* < 0.001, ***).

As the larvae were infected with *B. thailandensis*, deaths were recorded starting on day 1. At 10^2^ CFU *B. thailandensis,* 85% survival was observed on day 1, while 10^3^ CFU demonstrated 15% survival (Figure 10C,D). Increasing *B. thailandensis* CFUs to 10^4^ and 10^5^ further demonstrated 100% death rate on days 2 and 1, respectively (Figure 10E,F).

### 3.7. SFN Pre-Treatment Demonstrates Prophylactic Potential in G. mellonella Larvae Model

**Figure 11:**
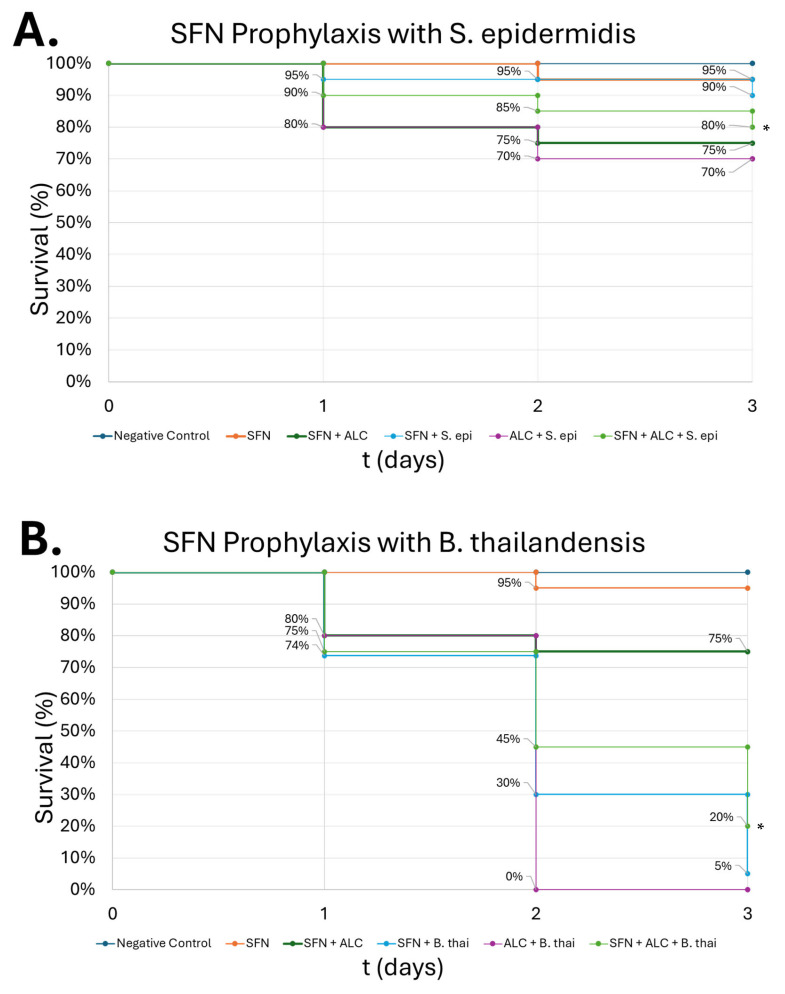
**Survival of *G. mellonella* larvae (n = 20) after SFN prophylaxis from alcohol and bacterial infection insults.** *S. epidermidis* at 10^5^ CFUs (**A**), *B. thailandensis* at 10^2^ CFUs (**B**). Negative control = no injection, Day zero = injection day. N = 20. Asterisks represent statistical differences between ALC + bacteria group and SFN + ALC + bacteria group (survival analysis, *p* < 0.05, *).

To replicate our in vitro live infection assay, we injected 100 µM SFN into the bottom right proleg of the larvae, followed by 0.08% (*v*/*v*) ALC in the bottom left proleg 3 h later. Either *S. epidermidis* or *B. thailandensis* was then injected into the larvae in the second cephalad pro leg one hour later with 10^5^ or 10^2^ CFU, respectively, based on infection dose–response curves. Larvae pretreated with SFN, then challenged with alcohol and co-cultured with live *S. epidermidis,* demonstrated 80% survival compared to 70% survival in larvae injected with ALC and *S. epidermidis* with no SFN on day 3. Groups injected with SFN and alcohol or SFN and *S. epidermidis* demonstrated 75 and 90% survival, respectively. Control groups demonstrated 95% or greater survival on day 3. Larvae pretreated with SFN, then challenged with alcohol and co-cultured with live *B. thailandensis,* demonstrated 20% survival compared to 0% survival in the group injected with alcohol and *B. thailandensis* with no SFN (Figure 11B). Groups injected with SFN and alcohol or SFN and *B. thailandensis* demonstrated 75 and 5% survival, respectively. Similarly, control groups demonstrated 95% or greater survival on day 3.

## 4. Discussion

Binge alcohol intoxication (BAI) in humans is defined as an elevated blood alcohol concentration to a minimum of 0.08% in a single episode [8]. BAI has a profound effect on innate immunity and is associated with impaired macrophage responses during an infection. The impairment of macrophage phagocytic ability increases the risk of opportunistic bacterial infections. Utilization of SFN as a prophylactic therapy to mitigate the deleterious effects of hazardous alcohol consumption on innate immunity remains to be explored. Furthermore, the cytoprotective effects of SFN as a pre-treatment to hazardous alcohol consumption in the context of a bacterial infection have not been quantified.

In the current study, murine MH-S alveolar macrophages and human THP-1 macrophages were used to model pulmonary inflammatory effects in two different species. THP-1 cells were differentiated from monocytes to macrophages as described previously. Murine MH-S cells treated with SFN for 24 h with doses below 20 μM resulted in cell viability of approximately 90%. However, human THP-1 cells treated with SFN doses below 10 μM for 24 h resulted in cell viability of approximately 90%. The downward and rightward shift in the THP-1 dose–response curve suggests an increased SFN potency with the human cell line (Figure 1). To test approximate equivalent doses in terms of cellular effects, 5 μM SFN was tested in subsequent experiments, as the cell viability was similar between cell lines. It is plausible that the differences in SFN cytotoxicity between species result from variations in metabolism. Nevertheless, these data indicate that 5 μM SFN is not cytotoxic to murine or human macrophage cells. Similarly, Eren et al. found that N9 microglial cells experienced no significant decrease in cell viability after 24 h exposure to SFN concentrations of 10 μM or less [35]. Tuttis et al. observed that human prostate cancer cells (DU145 and PC-3) treated with concentrations of 2, 4, and 8 μM of SFN for 24 h also showed no significant loss of cell viability [36]. Additionally, SFN in the 5–20 μM range may affect other cellular immune functions. Schwab et al. found in their study that human HT-29 colonocytes expressed mRNA for HBD-2, an endogenous antimicrobial peptide, in a dose-dependent manner starting at 5 μM up to 20 μM, expressing 3× greater mRNA levels compared to controls. Additionally, HBD-2 protein levels were 1.6× greater than untreated controls after being exposed to 20 μM SFN for 24 h of SFN exposure [37]. Myzak et al. noted that with a 10 μM oral gavage of SFN in APC^min^ mice, HDAC activity was decreased by 65%. There was also a significant 2-fold increase in H3 and H4 histone acetylation within the colonic mucosa after 6 h [38]. Likewise, in the current study, a significant effect in phagocytic function of macrophages after both three- and eight-hour incubation was measured. Taken together, these findings provide support for SFN doses below 10 μM being non-toxic and biologically relevant.

An alcohol binge is a single alcohol consumption event that brings blood alcohol concentration (BAC) to 0.08% or above. For most adults, this means having five or more drinks (male), or four or more drinks (female), in the space of nearly 2 h [39]. Moreira et al. provided an equation for the estimation of BAC, which would indicate that if a human male or female of average weight in the United States drank the minimum binge dose according to the NIAAA definition listed above, their blood alcohol would be 0.081% for males and 0.056% for females [40,41]. These benchmarks set an ideal minimum for studying binge alcohol type behavior in humans. However, in other experiments using an in vivo mouse model, an alcohol binge was defined as the administration of 5 g/kg body weight by gavage. The blood alcohol concentration of these mice reached nearly 400 mg/dL, which equates to a blood alcohol level of near 0.4%. It must be considered, however, that before administration of their “binge dose,” the mice in the study were observing a liquid diet meant to model chronic alcohol abuse and had pre-binge blood alcohol concentrations of near 180 mg/dL [42]. In the in vitro model, an alcohol dose of 220 mg/dL or 0.22% *v*/*v* was utilized to model hazardous alcohol consumption. Jimenez et al. noted that with concentrations of alcohol at 0.2% (*v*/*v*) and an exposure period of 8 h, MH-S macrophages had a 2.5-fold increase in intracellular bacterial survival, suggesting that 0.2% (*v*/*v*) alcohol is a dose that could dampen the innate immune response and set the stage for opportunistic infections. In the current study, various doses of alcohol were studied in both murine and human cells to optimize biologically relevant doses for the remainder of the study. Pursuant to these findings, we measured both human and murine macrophage viability when exposed to 0.2% (*v*/*v*) alcohol to be near 70–80%. The current study also noted significant increases in intracellular bacterial survival in Gram-positive and Gram-negative species when macrophages of both mice and humans were exposed to 0.2% (*v*/*v*) alcohol (Figure 3). These data suggest that 0.2% (*v*/*v*) alcohol is a dose capable of causing significant impairment to innate immune functions, yet preserves the viability of cells, indicating biological relevance. While this model allows for precise control of ethanol exposure, it also has limitations. Alcohol was introduced as a bolus dose to a simplified microenvironment to test the concentrations experienced during binge drinking. Yet, the in vitro model only partially reflects a human binge drinking episode, where alcohol would rise over time in a dynamic fashion as more is imbibed, metabolized and redistributed throughout tissues [43].

To test SFN as a pretreatment to hazardous alcohol exposure, murine and human tissue cells were pretreated with SFN for 24 h, followed by the removal of SFN treatment and exposure to the hazardous alcohol insult and bacterial challenge. Macrophage function was tested by measuring the viable intracellular survival of Gram-positive *S. epidermidis* and Gram-negative *B. thailandensis*. In both murine and human cell lines, alcohol exposure significantly impaired macrophage phagocytic ability (Figure 3). The alcohol-induced impairment served as a primary biological insult, allowing opportunistic pathogens to cause infection or prolonged bacterial survival. In fact, tens of thousands of deaths each year are attributed to alcohol-induced opportunistic infections [15]. In the current study, SFN pretreatment induced a 50-fold reduction in bacterial survival compared to alcohol only treated cells (Figure 4B). Treatment groups of both THP-1 and MH-S cell lines significantly decreased levels of bacteria compared with the ALC-only administered group. These data show that both *B. thailandensis*, a Gram-negative organism, and *S. epidermidis*, a Gram-positive organism, are susceptible to SFN-pretreated macrophages and thus, SFN could serve as a potential immunomodulating agent for defense against various opportunistic pathogens. Suganuma et al. demonstrated similar immunomodulatory results, where SFN-treated RAW 264.7 cells exhibited an increase in phagocytosis of 2 μM polystyrene beads through the inhibition of migration inhibitory factor (MIF) [30].

In addition to investigating macrophage function, immunomodulation was tested by measuring critical pro-inflammatory and anti-inflammatory cytokine levels (i.e., TNF-α, IL-10, and IFN-γ). TNF-α is a pro-inflammatory agent that regulates many facets of macrophage function and has been shown to be one of the most abundant early mediators in inflamed tissue [44]. IL-10 is a cytokine with potent anti-inflammatory properties, repressing the expression of prominent inflammatory cytokines [45]. IFN-γ is an autocrine cytokine that enhances a macrophage’s ability to mount an effective immune response, ranging from antigen presentation, autophagy of intracellular pathogens, and increased secretion of pro-inflammatory cytokines [46]. Schütze et al. demonstrated that macrophages exposed to increased levels of IFN-γ while co-stimulated with LPS reduced phagocytosis of *E. coli* 10-fold, warranting the measurement of IFN-γ in the presence of SFN [47].

When SFN was administered, a decrease in TNF-α levels along with constant levels of IL-10 in both control and treated populations was observed. In addition, IFN-γ was also measured at elevated levels in longer exposure to alcohol when not pretreated with SFN in both murine and human alveolar macrophages. It is plausible that there are two processes involved in decreasing levels of TNF-α and IFN-γ in the current study:

One, changes in TNF-α expression, may be the result of the inhibition of the nuclear factor kappa-light-chain-enhancer (NF-kB) pathway, in addition to SFN induced Nrf2 transcription modifications. Alcohol can induce ROS production through several mechanisms, such as cytochrome p450 family induction, alteration of metal levels in the body, and antioxidant elimination, leading to a state of oxidative stress [48]. This increased level of ROS eventually leads to the induction of the NF-kB pathway via inactivation of the IκB subunit, promoting translocation of NF-kB into the nucleus and promoting inflammation, notably with the production of TNF-α [49]. Although relative levels of TNF-α were lower in alcohol-exposed MH-S and THP-1 cells compared to the negative control, the TNF-α levels were still statistically significantly higher than SFN-treated cell lines alone. Similarly, Negi et al. found that treating Neuro2a cells with SFN decreased levels of NF-kB and abrogated high NF-kB levels induced by 30 mM glucose in Neuro2a cells when 0.5 and 1 mg/kg SFN were administered. Subsequent experiments measured TNF-α and IL-6 levels in both diabetic and normoglycemic rats that showed a significant reduction in both parameters when exposed to SFN at 0.5 mg/kg and 1 mg/kg [50]. Heiss et al. also demonstrated that prophylactic administration of 10–20 µM SFN to RAW 264.7 macrophages prior to LPS induction causes SFN to bind reversibly to Cys residues on NF-kB, inhibiting DNA binding of NF-kB [51]. Checker et al. additionally found that a 2 h pretreatment of 20 µM SFN in RAW 264.7 macrophages reduced secretion of IL-2, IL-4, IL-6, and IFN-γ in a concentration-dependent manner [52]. Likewise, sulforaphane has been reported to modulate membrane microdomains and redox-sensitive signaling events that are partly organized within lipid rafts. TLRs act as “adaptor” proteins with consequent IRAK phosphorylation and activation of NF-κB. Alcohol-induced alterations in lipid raft–dependent signaling may dysregulate inflammatory cytokine production and promote aberrant macrophage activation in the context of infection. Thus, targeting lipid rafts as a therapeutic approach may further elucidate the therapeutic mechanism of SFN [53].

Furthermore, Gasparello et al. showed the therapeutic potential of SFN as an adjuvant therapy in COVID-19 by demonstrating the ability of SFN to significantly reduce the production of proinflammatory cytokines IL-1β and IL-8 as well as reduce the expression of the inflammatory transcription factor NF-κB in bronchial epithelial cells [54]. These data demonstrate the potential for SFN as an immunotherapy to mitigate infection beyond bacteria to include viruses. Interestingly, Saez et al. discovered that SFN pretreatment of HeLa cells for 24 h prior to infection with *Chlamydia trachomatis* for 24 h corresponded with a significantly increased number and size of intracellular inclusion bodies, indicating a worsening of the infection. Given that SFN is a known inducer of antioxidant genes and *C. trachomatis* is an obligatory intracellular bacterium, they hypothesized that an increased quantity of intracellular ROS may be a mechanism by which the cells combat Chlamydia species after they additionally found that inducing intracellular ROS decreased infection burden [55,56]. These findings together point to the fact that although SFN may have broad-reaching potential for the improvement of immune function in Gram-negative, Gram-positive, and even viral infections, there may be some pathogens and associated infectious mechanisms in which SFN may not be a beneficial agent. The current study reinforces the therapeutic potential of SFN as a possible prophylactic measure to prevent some serious nosocomial infections.

**Figure 12:**
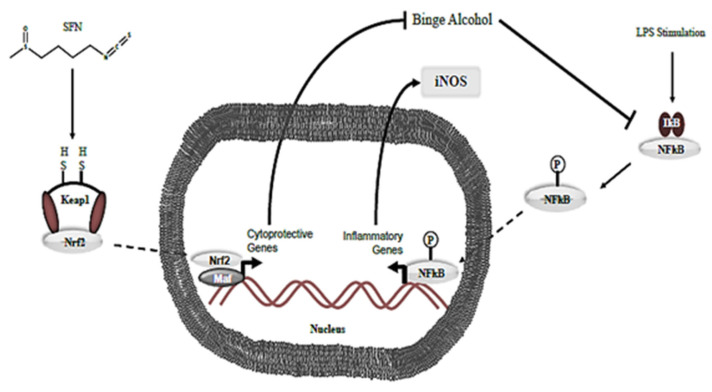
**Hypothesis for the SFN mechanism to counteract the effects of binge alcohol use.** SFN enables the detachment of Keap-1 (endogenous cytoplasmic inhibitor) from the Nrf2 transcription factor. Subsequent Nrf2 translocation to the nucleus allows for the transcription of cytoprotective genes against alcohol insult. Thus, SFN modulates the inflammatory response through means such as NF-kB.

Second, additional experiments have demonstrated increased nuclear translocation of Nrf2 and Nrf2 expression due to SFN treatment [57]. Given Nrf2’s role in transcription of several anti-oxidation-related genes, this could be another possible explanation for the decrease in levels of TNF-α. Previous studies have shown that SFN increases the expression of Nrf2, an important antioxidative transcription factor. To validate the expression of Nrf2 in SFN-treated macrophages in the current study, Nrf2 gene expression was measured. A subsequent 3-fold increase in Nrf2 levels was observed when compared to an untreated control. To mimic the environment of Gram-negative bacteria, LPS was added, and a 12-fold increase in Nrf2 expression was noted (Supplementary Figure S1). These data illustrate that SFN is acting, at least in part, through its upregulation of Nrf2 and continue to suggest that SFN can effectively serve as a potential immuno-treatment to prevent opportunistic infections due to dysfunctional immune response, as seen in binge alcohol intoxication (Figure 12).

The therapeutic potential of SFN was extended beyond an in vitro cell culture model to include an in vivo *G. mellonella* model. Colloquially known as waxworms, these species possess a hemolymph system that is like that of the human innate immune system. These larvae are especially useful in studying the relationship between toxins, infections, and the innate immune system, and have been used to study more prominent bacteria and toxins. Several studies have explored the use of SFN within the larvae that focus on the direct interaction of SFN with *Shigella* and *Vibrio* spp., not on SFN’s potential ability to bolster the immune system [58,59]. Additionally, there is limited literature on the effects of alcohol on the larvae, limited to the threshold dose for alcohol if used as a solvent. Suay-Garcia et al. demonstrated that alcohol concentrations up to 30% were considered safe to use within the larvae [60]. Finally, *B. thailandensis* has been used in the past in a *G. mellonella* model, but there is no literature on the use of *S. epidermidis* in the same model [61]. Ultimately, the *G. mellonella* model provides a cheaper yet promising alternative platform to evaluate how alcohol exposure and pathogen challenge interact with SFN prophylaxis in an in vivo model.

In the current study, waxworms tolerated increasing doses of SFN up to 100 μM, with survivability up to 80% at 50 μM of SFN (Figure 6). This is consistent with studies by Nowicki et al. and Krause et al., which used concentrations up to 25 mg/kg in the larvae that were generally well tolerated [58,59]. A total of 100 μM SFN was used for the final study to minimize SFN degradation within the larvae before introducing alcohol. Next, the larvae were injected with increasing concentrations of alcohol. Interestingly, the larvae showed increasing tolerance to alcohol in a dose-dependent manner, showing greatest lethality at 0.08% (*v*/*v*) (Figure 7C). The typical diet of waxworms contains raw beeswax, which may offer some explanation for this tolerance of alcohol at higher concentrations. A similar metabolic trait is present in the Oriental hornet *Vespa orientalis,* which feeds on fermented carbohydrates on top of other insects, creating an adaptation to permit chronic consumption of alcohol in concentrations up to 80% [62]. Waxworms may share this type of alcohol tolerance. Lower concentrations of alcohol may not reach the required threshold to induce protein expression for alcohol metabolism, which could account for the lethality observed in these experiments. Alcohol concentrations of 0.08% (*v*/*v*) were used for future experiments due to the consistent demonstration of the greatest lethal effect on *G. mellonella*. This concentration also represents a similar binge-alcohol concentration in humans and helps solidify the model as an analog for human physiology.

Gram-positive *S. epidermidis* and Gram-negative *B. thailandensis* were inoculated in the larvae to determine the LD_50_ of the pathogens. In the *S. epidermidis* injections, the survival curve exhibits a dose-dependent lethality on the larvae, showing the greatest lethality at 10^5^ CFU at 50% on day 3 (Figure 9C). *B. thailandensis* injections demonstrated greater lethality compared to *S. epidermidis*, due to the greater virulence typically observed among Gram-negative bacteria compared to Gram-positive organisms. An LD_50_ was observed in concentrations as low as 10^2^ CFU, for which that group had 85% lethality by the end of the observation period (Figure 10C). Wand et al. produced similar results, where different strains of *B. thailandensis* at 10^2^ CFU consistently produced mortality within the larvae between 50 and 80% after a 24 h observation period [63]. The dose-dependent lethality observed with *S. epidermidis* and *B. thailandensis* affirms prior findings that *G. mellonella* serves as a reliable infection model capable of distinguishing differences in bacterial virulence [32,64]. The more lethal outcomes associated with *B. thailandensis* are in line with known distinctions between Gram-negative and Gram-positive organisms, particularly the role of lipopolysaccharides in Gram-negative bacteria, which can amplify host immune responses and resistance to clearance [17].

SFN pretreatment presented partial protection against the combined insult of alcohol and infection, improving survivability relative to groups that did not receive the SFN pretreatment (Figure 11). While the survival rate was not returned to baseline levels, this outcome is consistent with mammalian models where SFN enhanced macrophage anti-bacterial destruction and reduced inflammatory dysregulation, even if it did not completely reverse the effects of the pathogen [32]. These data support the translational relevance of our *G. mellonella* findings and suggest that SFN’s protective effects are robust across species. These findings emphasize the promise of SFN as a prophylactic intervention that mitigates alcohol related immune compromise. Building on this work, future studies should integrate transcriptomic or proteomic profiling in *G. mellonella,* additional microenvironment studies to evaluate changes in ROS, evaluate a broader spectrum of pathogens, and extend validation to mammalian systems. By doing this, we can better understand the mechanism by which SFN supports host defense and further establish the *G. mellonella* model’s potential.

## 5. Conclusions

In this study, we showed for the first time that in murine MH-S cells and human THP-1 cells, SFN pre-treatment prevents intracellular pathogen killing dysfunction after hazardous alcohol exposure. Moreover, the results indicate that (1) the dose-dependent effects of SFN are consistent across different species, (2) SFN pre-treatment prevents alcohol-induced macrophage dysfunction, and (3) SFN cytoprotective effects are independent of Gram-negative or Gram-positive bacterial infection. Additionally, the results also indicate that SFN prophylaxis improves survivability in *G. mellonella* larvae, increasing confidence in the ability of SFN to bolster innate immune defenses in vivo. Taken together, SFN has the potential to serve as an immuno-modulating agent by preserving important innate phagocytic capability in first-line defense cells such as alveolar macrophages during phagocytosis of both typical Gram-positive and Gram-negative organisms.

## Data Availability

The data presented in this study are available on request from the corresponding author due to privacy requirements.

## Supplementary Materials

The following supporting information can be downloaded at: https://www.mdpi.com/article/10.3390/medicines13010008/s1, Supplementary Figure S1. Nrf2 expression after exposure to various combinations of SFN, Alcohol (ALC), and LPS. Protein expression of Nrf2 in alveolar macrophage (AMs). Cells pre-treated with SFN, then Alcohol (ALC) or LPS. AM cells were treated for 24 h with SFN (5 µM) before challenging cells with 0 or 0.2% (*v*/*v*) alcohol for 3 or 8 h. To establish a baseline, cells were treated with 0.2% (*v*/*v*) alcohol for 3 or 8 h without SFN pre-treatment. Additional cells were treated with LPS concentrations for 8 h or pre-treated with SFN and challenged with LPS for 3 or 8 h. Data are expressed as means ± SEM. Statistically significant results by one-way ANOVA are indicated by asterisks *** *p* < 0.001 by two-way ANOVA.
