# Supplementary figures and images for: Sulforaphane Pre-Treatment Improves Alveolar Macrophage Killing After Alcohol-Induced Phagocytic Dysfunction In Vitro and in Galleria mellonella Larvae
Source: Medicines (Basel). 2026 Feb 19;13(1):8. doi: 10.3390/medicines13010008 (PMC13028045; doi:10.3390/medicines13010008)

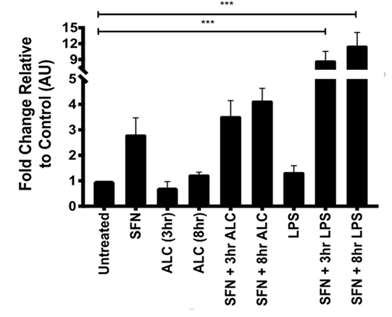

Supplement: Supplementary file 1 [file medicines-13-00008-s001.zip › medicines-3969314-supplementary.tif]
